# Association between smoking and the risk of acute mountain sickness: a meta-analysis of observational studies

**DOI:** 10.1186/s40779-016-0108-z

**Published:** 2016-12-08

**Authors:** Chen Xu, Hong-Xiang Lu, Yu-Xiao Wang, Yu Chen, Sheng-hong Yang, Yong-Jun Luo

**Affiliations:** 1 Department of Military Medical Geography, Third Military Medical University, Chongqing, 400038 China; 2 Battalion 5 of Cadet Brigade, Third Military Medical University, Chongqing, 400038 China; 3 Key Laboratory of High Altitude Environmental Medicine (Ministry of Education), Third Military Medical University, Chongqing, 400038 China; 4 Mountain Sickness Research Institute, 18th Hospital of PLA, Yecheng, Xinjiang 844900 China

**Keywords:** Smoking, Acute mountain sickness, Association, High altitude, Meta-analysis, Risk factor

## Abstract

**Background:**

People rapidly ascending to high altitudes (>2500 m) may suffer from acute mountain sickness (AMS). The association between smoking and AMS risk remains unclear. Therefore, we performed a meta-analysis to evaluate the association between smoking and AMS risk.

**Methods:**

The association between smoking and AMS risk was determined according to predefined criteria established by our team. Meta-analysis was conducted according to the PRISMA guidelines. We included all relevant studies listed in the PubMed and Embase databases as of September 2015 in this meta-analysis and performed systemic searches using the terms “smoking”, “acute mountain sickness” and “risk factor”. The included studies were required to provide clear explanations regarding their definitions of smoking, the final altitudes reached by their participants and the diagnostic criteria used to diagnose AMS. Odds ratios (*ORs*) were used to evaluate the association between smoking and AMS risk across the studies, and the *Q* statistic was used to test *OR* heterogeneity, which was considered significant when *P* < 0.05. We also computed 95% confidence intervals (CIs). Data extracted from the articles were analyzed with Review Manager 5.3 (Cochrane Collaboration, Oxford, UK).

**Results:**

We used seven case-control studies including 694 smoking patients and 1986 non-smoking controls to analyze the association between smoking and AMS risk. We observed a significant association between AMS and smoking (*OR* = 0.71, 95% CI 0.52–0.96, *P* = 0.03).

**Conclusions:**

We determined that smoking may protect against AMS development. However, we do not advise smoking to prevent AMS. More studies are necessary to confirm the role of smoking in AMS risk.

## Background

People rapidly ascending to high altitudes (>2500 m) may suffer from acute mountain sickness (AMS), an illness characterized by symptoms such as headache, loss of appetite, nausea and vomiting, dizziness, fatigue and sleep disturbances [1, 2]. Not all symptoms must be present for an AMS diagnosis. In some severe cases, AMS may evolve into high-altitude cerebral edema (HACE) or high-altitude pulmonary edema (HAPE), conditions that may threaten patient health and well-being [3]. AMS symptoms usually occur during the first night at high altitude. AMS remission typically occurs a few days later, once affected individuals have adapted to the new altitude [4]. The Lake Louise Scoring System is a standard commonly used for diagnosing AMS that was established at the 1991 International Hypoxia Symposium [5, 6]. Other standards, including the Environmental Symptom Questionnaire (ESQ), the AMS Symptom Questionnaire, and the General High-Altitude Questionnaire, have also been used [7].

Despite decades of research, the pathophysiological mechanisms underlying AMS development remain poorly understood. Previous studies have attempted to identify risk factors that can be used to predict AMS susceptibility. A substantial number of studies have shown that age [8, 9], body mass index (BMI) [9, 10], arterial oxygen saturation [11], sleep quality [12] and gender [13, 14] are correlated with AMS susceptibility. However, other studies have found that these factors do not play a role in AMS development [15]. MacInnis et al. [16] reviewed the relationships between various genetic factors and AMS in 2010 and found no genetic factors that can reliably predict AMS. Therefore, searching for factors that are related to AMS development remains necessary. Wu et al. [17] reported that the incidence of AMS was lower among smokers than among non-smokers and that smokers exhibited lower AMS scores than non-smokers. A similar conclusion was reached by Song et al. [18]. In addition to the above-mentioned reports, other studies suggest that smoking is a protective factor for AMS. For example, You et al. [19] found that the incidence of AMS among smokers (26.1%) was significantly lower than that among non-smokers (47.2%). However, studies conducted by Ziaee et al. [15], Gaillard et al. [20] and Vinnikov et al. [21] suggested that there was no significant correlation between AMS and smoking habits.

Recently, Vinnikov et al. [22] performed a meta-analysis showing that smoking was not significantly associated with AMS. Different detailed method might get different conclusions. Based on multiple reviews of several articles, we determined that some studies did not use common standards for diagnosing AMS and that other studies did not provide concrete data regarding the incidence of AMS among smokers and non-smokers. Additionally, several studies used non-rigorous inclusion criteria, which likely influenced their results; thus, we used strict inclusion and exclusion criteria in this analysis, which we performed with the aim of investigating the association between smoking and AMS risk to add to the knowledge base regarding this issue.

## Methods

### Search strategy

The PRISMA guidelines were used to screen eligible studies. We searched the English-language literature regarding smoking and AMS that was published before September 2015 using the PubMed and Embase databases. Our search strategy included the following key words: 1) acute mountain sickness (AMS), 2) smoking and 3) risk factor. We did not include abstracts or unpublished reports in the analysis. If the same subjects were published in different publications, the study with the more comprehensive analysis was extracted. Two investigators extracted the data independently; in the event of disagreement, consensus was reached *via* discussion.

### Inclusion and exclusion criteria

The following studies were included in the analysis: 1) including smoking and non-smoking data, 2) regarding AMS, 3) providing original data regarding the numbers of smokers and non-smokers and AMS occurrence, 4) using high-altitude exposure definitions similar to those of other studies, 5) original data were obtained at an altitude above 3000 m, and 6) using clear and common diagnostic criteria for AMS.

The following studies were excluded: 1) lacking specific data regarding smoking and non-smoking, 2) studies with insufficient information about the number of AMS cases, 3) studies that did not mention the direct relationship between smoking and AMS, and 4) studies that did not use or discuss common diagnostic criteria for AMS.

### Data extraction and synthesis

For each study, the first author, year of publication, altitude, and numbers of smokers and non-smokers with AMS were recorded. If one study provided multiple detailed data sets related to smoking and AMS, all of these data were recorded.

### Statistical analysis

The association between smoking and AMS was assessed in this meta-analysis. To determine the association between smoking and AMS, we performed a comparison of the prevalences of AMS in smokers and non-smokers. Odds ratios (ORs) were used to evaluate the association between smoking and AMS risk across the studies. The *Q* statistic was used to test *OR* heterogeneity, which was considered significant when *P* < 0.05. The *I* ^2^ statistic was also calculated to quantitatively assess inconsistency. We also computed 95% confidence intervals (CIs). Additionally, if there was significant heterogeneity, we used a random effects model to analyze the study. If there was no heterogeneity, we used a fixed effects model [2, 23–25]. We also performed Begg’s and Egger’s tests when significant differences (*P* < 0.05) were present to determine if publication bias was also present.

## Results

### Description of studies

**Fig. 1:**
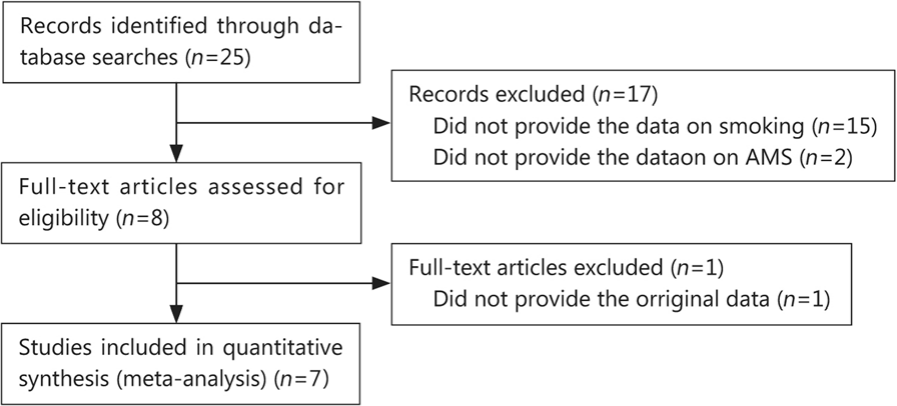
Selection flow chart

We initially retrieved 25 articles from PubMed and Embase. Based on the above-mentioned pre-specified exclusion criteria, we re-evaluated all of the studies. We found that 15 articles lacked concrete data pertaining to smoking and non-smoking groups, 2 articles lacked AMS diagnostic criteria, 1 article did not provide the original data. Seven articles were ultimately included according to our exclusion and inclusion criteria (Fig. 1). The studies by Schneider et al. [4] and Mairer et al. [28] each provided two data sets; the subjects in the two groups were not repeat subjects, so we considered the studies in question to be independent studies, which were identified using the indicated key words. The following search terms were used: (acute mountain sickness or AMS or high altitude) and (smoking) and (risk factor). We included a total of seven articles including 694 smoking subjects and 1986 non-smoking controls in our meta-analysis. One report used the Environmental Symptom Questionnaire (ESQ), while the other reports used the Lake Louise Scoring (LLS) System. The extracted data are presented in Table 1.

**Table 1:** Extracted data characteristics

Research	Altitude (m)	Sample size		AMS		Normal		Diagnosis of AMS with cut-off value	Age (mean)		BMI (mean)		Sex (m/f)	
		Smoking	Non-smoking	Smoking	Non-smoking	Smoking	Non-smoking		AMS	Normal	AMS	Normal	AMS	Normal
Ren, 2015 [14]	4300	16	64	3	32	13	32	LLS > 3	38.1	38.6	24.7	26.2	9/26	22/23
Wu, 2012 [17]	4525	182	200	71	102	111	98	LLS >3 OR LLS >4						
You, 2012 [19]	4300	138	176	36	83	102	93	LLS > 4	20.08	20.25	21.31	21.47	119/0	195/0
MacInnis, 2013 [26]	4380	147	344	42	125	105	219	LLS ≥ 3					100/67	244/80
Mairer, 2010(a) [28]	3454	9	66	4	26	5	40	LLS ≥ 4	35.1	34.5	23.4	23.3	25/5	39/6
Mairer, 2010(b) [28]	3817	13	67	3	25	10	42		36.2	38.1	23.5	22.8	22/6	41/11
Mairer, 2009 [27]	3500	61	370	12	58	49	312	LLS ≥ 4	38.4	37.2	23.7	23.3	50/20	266/86
Schneider, 2002(a) [4]	4559	56	331	16	103	40	228	ESQ ≥ 0.70	38.2		22.6		314/73	
Schneider, 2002(b) [4]	4559	72	368	21	99	51	269		37.0		22.7		359/81	

Mairer (2010) [28] reported two studies (2010a and 2010b) that were independent. Schneider (2002) [4] also published two studies (2002a and 2002b) that were independent. Schneider (2002) [4] used the Environmental Symptom Questionnaire (ESQ) to diagnose AMS using an ESQ cut-off value of 0.70, which corresponded to a Lake Louise Score (LLS) of 4. Wu (2012) [17] did not provide original data regarding age, gender or BMI. MacInnis (2013) [26] did not provide clear data regarding age or BMI. Schneider (2002) [4] provided only data regarding the total population of subjects. In addition, we analyzed how the studies defined smoking and found that only one article (Wu (2012) [17]) provided the following clear definition of smoking: “A smoker was someone who smoked 10 or more cigarettes/day for >6 months”

### Meta-analysis results

**Fig. 2:**
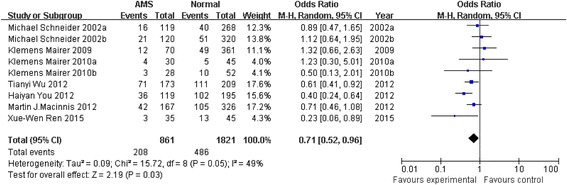
Forest plot of the relationship between AMS and smoking. The disease occurrence (odds ratio) summary is displayed in the comparison between smokers and non-smokers, and 95% confidence intervals are shown on the extreme *left* and *right*. The incidence of AMS is expressed as the number of events

Seven articles [4, 14, 17, 19, 26–28] including 694 smokers and 1986 non-smoking controls were included in our study. We analyzed these 7 articles and found that smoking could reduce the prevalence of AMS. This allowed us to use RevMan 5.0 to analyze the data. Heterogeneity analysis showed that df = 8, *P* = 0.05, and *I* ^2^ = 49% (Fig. 2), indicating that the 7 articles were not homogeneous in nature. Smoking could reduce the prevalence of AMS, with an *OR* of 0.71 (95% CI 0.52–0.96). The test for overall effect, *Z* = 2.19 (*P* = 0.03), clearly showed that smoking was lower in the AMS group than in the control group; this difference was statistically significant.

### Publication bias analysis

**Fig. 3:**
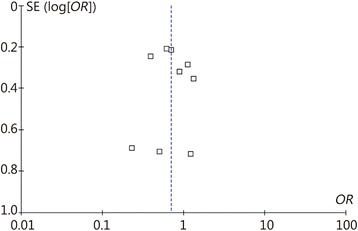
Funnel plot of publication bias. According to the funnel plot, no publication bias was present. Each point represents a separate report providing information regarding the indicated association

We constructed a funnel plot to determine if publication bias was present. Based on the symmetry of the funnel plot, as shown in Fig. 3, we postulate that no publication bias was present in the articles included in our analysis.

## Discussion

In our study, we analyzed a total of 694 smokers and 1986 non-smoking controls from seven published articles. To our knowledge, prior our study, only one meta-analysis explored the association between smoking and AMS and found that smoking was not significantly associated with AMS [22]. Different selection criteria may lead to different results; we used strict inclusion and exclusion criteria and obtained results that contrasted with those of the previous study.

Our meta-analysis results contrast with the traditional opinion that smokers are more like to develop AMS than non-smokers. The following study findings may explain why smoking protects people from AMS. First, Sanchez del Rio *et al*. [29] found that nitric oxide (NO) can irritate and discharge the nerve fibers constituting the trigeminovascular system, resulting in high-altitude headaches. Moreover, NO increases blood-brain barrier permeability, which may also cause headaches [30]. Studies have shown that endothelial function can be impaired by small amounts of oxygen free radicals in smokers, resulting in decreased NO synthesis and increased NO decomposition [31]. Therefore, lower brain NO levels may, to a certain extent, protect smokers from developing headaches [17]. Second, periodic breathing, a breathing pattern characterized by waxing and waning respiration, may play a major role in sleep disturbances at high altitude [32]. Studies have shown that breathing regulation may be influenced by NO and carbon monoxide (CO) [33, 34]. Because of higher CO and NO levels, smokers experience fewer sudden bursts of breathing and subsequent arousals from sleep [32]. Therefore, smokers usually exhibit more stable breathing and sleep better than non-smokers [17]. Third, previous experiments have demonstrated that CO can simultaneously increase vascular smooth muscle apoptosis and decrease vascular smooth muscle cell proliferation, which is thought to be beneficial with respect to pulmonary arterial pressure (PAP) [35]. Reports have also shown that CO reverses established pulmonary arterial hypertension (PAH). Thus, PAP can be decreased by increases in CO inhalation, which protect against the development of hypoxia. Therefore, higher CO levels in smokers prevent the development of vascular disorders and may thus decrease the risk of AMS. Fourth, smokers exhale more CO, which can bind to hemoglobin (Hb) and reduce its oxygen carrying potential [36]. In contrast, You *et al*. [19] reported that smokers exhale more CO, but the occurrence of AMS was lower in non-smokers than in smokers; therefore, the fraction of exhaled CO may protect against AMS within a particular range. Fifth, Baumgartner *et al*. [37] reported that AMS was related to cerebral blood flow. Another study reported that the cerebral blood flow velocities of smokers were lower than those of non-smokers [18], resulting in a lower incidence of headaches and a lower incidence of AMS. Finally, the present study clearly determined that cigarette smoking is one of the major causes of polycythemia [38]. Moderate increases in Hb levels have been shown to be beneficial with respect to blood oxygen-carrying capacity at high altitude in smokers, as these increases may correct reductions in arterial oxygen saturation, which have been shown to be closely associated with AMS [39].

While smoking appears decrease the risk of AMS development, we do not advise smoking to prevent AMS. Smoking cigarettes increases the risk of developing cardiorespiratory diseases, as well as other diseases, including cancer [17]. However, further research regarding the mechanisms underlying this phenomenon may enable us to identify other preventive measures that have the same beneficial effects as smoking. In any event, the best way to prevent AMS is by making a gradual ascent and allowing sufficient time for acclimatization to higher altitudes [32].

There were several limitations to our study. First, only a relatively small number of studies were eligible for inclusion in our analysis, which may have limited our results. Second, five studies did not attempt to document CO/NO levels. Third, patients were diagnosed with AMS by different observers, which might have influenced the outcomes of the studies. Fourth, we did not analyze other factors associated with AMS, such as age, gender, and BMI.

## Conclusion

We conclude that smoking may protect against AMS. However, more case-control studies are necessary to clarify the role of smoking in AMS risk further.

## Abbreviations

AMS: Acute mountain sickness
BMI: Body mass index
CI: Confidence interval
CO: Carbon monoxide
ESQ: Environmental symptom questionnaire
HACE: High-altitude cerebral edema
HAPE: High-altitude pulmonary edema
Hb: Hemoglobin
LLS: Lake Louise Scoring
NO: Nitric oxide
OR: Odds ratio
PAH: Pulmonary arterial hypertension
PAP: Pulmonary arterial pressure
